# Comparison of hemostatic dressings for superficial wounds using a new spectrophotometric coagulation assay

**DOI:** 10.1186/s12967-015-0740-5

**Published:** 2015-11-30

**Authors:** Julian-Dario Rembe, Julia K. Böhm, Carolin Fromm-Dornieden, Nadine Schäfer, Marc Maegele, Matthias Fröhlich, Ewa K. Stuermer

**Affiliations:** Institute for Research in Operative Medicine (IFOM), Witten/Herdecke University, Ostmerheimer Str. 200, 51109 Cologne, Germany; Department of Traumatology, Orthopaedic Surgery and Sports Traumatology, Cologne-Merheim Medical Centre (CMMC), Witten/Herdecke University, Campus Cologne-Merheim, Ostmerheimer Str. 200, 51109 Cologne, Germany

**Keywords:** Superficial wound dressing, Hemostasis, Hemostatic agents, Absorption, Oral anticoagulation

## Abstract

**Background:**

Due to demographical changes the number of elderly patients depending on oral anticoagulation is expected to rise. Prolonged bleeding times in case of traumatic injuries represent the drawback of these medications, not only in major trauma, but also in superficial wounds. Therefore, dressings capable of accelerating coagulation onset and shortening bleeding times are desirable for these patients.

**Methods:**

The hemostatic potential and physical properties of different types of superficial wound dressings (standard wound pad, two alginates, chitosan, collagen (Lyostypt^®^), oxidized cellulose, and QuikClot^®^) were assessed in vitro. For this purpose the clotting times of blood under the influence of the named hemostatics from healthy volunteers were compared with Marcumar^®^ or ASS^®^ treated patients. For that, a newly developed coagulation assay based on spectrophotometric extinction measurements of thrombin activity was used.

**Results:**

The fastest coagulation onset was observed for oxidized cellulose (Ø 2.47 min), Lantor alginate-l (Ø 2.50 min) and QuikClot^®^ (Ø 3.01 min). Chitosan (Ø 5.32 min) and the collagen Lyostypt^®^ (Ø 7.59 min) induced clotting comparatively late. Regarding physical parameters, QuikClot^®^ showed the lowest absorption capacity and speed while chitosan and both alginates achieved the highest. While oxidized cellulose displayed the best clotting times, unfortunately it also revealed low absorption capacity.

**Conclusions:**

All tested specimens seem to induce clotting independently from the administered type of oral anticoagulant, providing the possibility to neglect the disadvantage in clotting times arising from anticoagulation on a local basis. QuikClot^®^, oxidized cellulose and unexpectedly alginate-l were superior to chitosan and Lyostypt^®^. Due to its additional well-known positive effect on wound healing alginate-l should be considered for further investigations.

## Background

Diseases such as arteriosclerosis, atrial fibrillation (AF), arterial hypertension and diabetes mellitus increase the risk for cardiac diseases and thromboembolic events. In line with an aging population, this is expected to lead to an increased number of patients requiring oral anticoagulants (OACs) or antiplatelet therapies (APT) in the future [1]. The prevalence of atrial fibrillation is about 2 % in the overall population and will prospectively rise continuously to about 10 % in people over the age of 80 years [2, 3]. Already, a large number of humans over 60 years depend on drugs like acetylsalicylic acid (ASS^®^), P2Y12 antagonists (e.g. clopidogrel, ticagrelor), vitamin K antagonists (VKA, e.g. Marcumar^®^), new oral anticoagulants (NOACs, e.g. rivaroxaban) or even a combined anticoagulation therapy (ACT) to treat and prevent thromboembolism, myocardial infarction and strokes [4, 5]. Unfortunately, these medications are also responsible for bleeding complications after traumatic injuries or surgical procedures [6, 7].

In the last two decades, regimes regarding the perioperative management of ACT were developed [8, 9]. These “bridging” regimes exist for each kind of OAC, APT or their combinations [10] and are widely established for planed interventions and surgery. However, they are inapplicable in case of acute trauma with major hemorrhage. The iatrogenic coagulopathy can be addressed to a certain degree by performing systemic damage control resuscitation via transfusion [11] or locally by hemostatic dressings combined with local pressure as used in emergency care [12]. Still, surgical procedures and hemorrhage control of patients receiving ACT is challenging.

Furthermore, minor bleedings of trivial wounds also represent a daily challenge for patients with ACT. A simple incisional wound under ACT can bleed up to 20 min or longer if untreated (personal experience). The decelerated clotting which leads to discomfort and stress to the wounded person may not necessarily represent a major threat demanding an acute intervention. But as there is a rising number of persons concerned, these minor injuries become more troublesome and a reoccurring problem for the individual. In addition, it has been shown that long-term intake of OAC is associated with reduced quality of life, including bleeding complications and the fear for such [13]. It can be assumed that repeated injuries with prolonged bleeding times, especially in case of impaired wound healing resulting from comorbidities or reopening, further foster a reduction in quality of life. Furthermore, it needs to be considered that coagulation represents the first phase of wound healing [14]. Therefore, a prolonged primary coagulation could impair the healing process and increase the risk of wound chronification even in small wounds. Hemostatic dressings, like QuikClot^®^, used in emergency care demonstrate a useful tool to control bleeding faster [15, 16] and thus, should be transferred to daily practice.

In view of the outlined complications and quality of life limitations for patients under ACT this study aimed to evaluate different new as well as established kinds of wound contact material regarding their capability of accelerating coagulation times in vitro. Additionally, physical parameters relevant for a wound dressing like weight, absorption capacity and speed, tissue adhesion as well as pH and temperature influence of all specimens were assessed. Based on these results the most suitable hemostatic superficial wound dressings were identified for future investigations. So far, the established dressings are only used in emergency care and warfare medicine but may represent a promising option for daily clinical practice and home care to handle excessively bleeding superficial wounds. To determine their potential in accelerating blood coagulation a novel in vitro method based on a chromogenic assay was used.

## Methods

### Specimen and physical properties

**Fig. 1:**
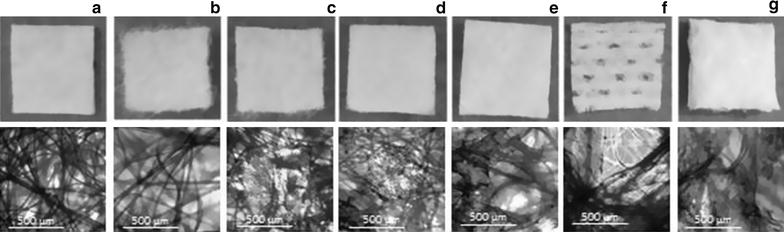
Macro- and microscopic pictures of the investigated specimens (1 cm^2^). **a** standard wound pad; **b** DRACO alginate (-d); **c** lantor alginate (-l); **d** oxidized cellulose; **e** chitosan; **f** QuikClot^®^; **g** Lyostypt^®^. (Magnification: 1:1 & 1:100)

Seven different wound pads (Fig. 1; Table 1) were evaluated in respect to their potential to accelerate coagulation. Additionally, physical properties such as weight, absorption capacity and time needed to absorb 50 µl of whole blood were determined for all pads. For these analyses, specimens were cut into pieces of 1 cm^2^ (0.5 cm × 2 cm for spectrophotometric measurements, 1 cm × 1 cm for physical property tests). Only Quikclot^®^ was prepared in pieces of 0.5 cm × 4 cm and subsequently laid double due to its thinness resulting in the same size (0.5 cm × 2 cm) as all tested pads. In case of using QuikClot^®^ as a plaster pad the double layer would be its reasonable application.

**Table 1:** Investigated dressings

	Manufacturer	Material	Mechanism of action
Wound pad	Dr. Ausbüttel & Co. (DRACO), Witten, Germany	Viscose, polypropylene/polyethylene nonwoven with microperforated PE film	Control dressing (none expected)
Alginate-d	Dr. Ausbüttel & Co. (DRACO), Witten, Germany	Carboxymethyl cellulose containing calcium alginate	Release of Ca^2^ ions induces activation of platelets and plasmatic coagulation [17]
Alginate-l	Lantor GmbH, Haibach, Germany	Viscose, polyolefin and polyester containing calcium alginate	See ‘Alginate-d’
Oxidized cellulose	Resintex Industriale s.r.l., Pieve Emanuele, Italy	Oxidized cellulose	Unknown; presumably platelet activation [18]
Chitosan	Heppe Medical Chitosan GmbH, Halle (Saale), Germany	Polysaccharide derived from chitin	Unknown; presumably platelet activation [19]
QuikClot^®^	Z-MEDICA, LLC, Wallington, USA	Kaolin-impregnated gauze	Unknown; presumably concentration of procoagulant factors due to water absorption and Ca^2+^ release [20]
Lyostypt^®^	B. Braun Melsungen AG, Melsungen, Germany	Bovine collagen derived fleece	Platelet adhesion and activation of coagulation factor XII [21]

Data on manufacturer, material and mechanism of action

Dry weight of all specimen (1 cm^2^ pieces) were determined by measuring three samples using a fine scale (ALJ 160-4M; Kern & Sohn GmbH, Balingen-Fromern, Germany) and mean ± SD were calculated. Subsequently, fresh whole blood from healthy volunteers was stepwise administered to the pads until saturation and maximum absorption capacity in µl/cm^2^ was determined.

To determine the absorption speed of the investigated dressings (in seconds) 50 µl of fresh whole blood was applied to the samples unsing a 100 µl pipette (Eppendorf AG, Hamburg, Germany) and time needed to absorb the blood was measured by stopwatch. All measurements were performed in triplicates.

### Tissue adhesion, pH and temperature measurement

A 1.5 cm × 3 cm piece of each tested pad was placed on fresh porcine skin [22] and exposed to 50 µl of fresh whole blood from untreated, healthy volunteers for 24 h. Within this period the blood coagulated and agglutinated with the pads and thereby attached to the porcine skin. The adhesive strength of each pad to the porcine skin was determined with a digital force gauge (Sauter, Balingen, Germany) in a peak-tension mode with a speed of 0.5 mm/s.

The pH and temperature differences between the wound pads submerged in human fresh frozen plasma (FFP) of untreated, healthy volunteers were determined at baseline, after 15, 30, 60 and 90 min. Changes were measured by the inoLab^®^ pH 720 (WWT, Weilheim, Germany).

### Subject groups and sample collection

The study was conducted with 20 untreated volunteers (group A), 20 patients treated with the coumarin derivative Marcumar^®^ (group B) and 30 patients with daily intake of acetylsalicylic acid (ASS^®^; group C). For untreated volunteers no intake of anticoagulants in the last 10 days was mandatory.

In advance, ethical approval was obtained by the ethics committee (Witten/Herdecke University, Germany; FN: 16/2014). The inclusion criteria for all groups were as followed: Caucasian and age ≥50 years for homogeneity of the study group, as well as the signed informed consent. In the Marcumar^®^ group an international normalized ratio (INR) of >1.5 and ≤3.5 was defined as additional inclusion criteria while in the ASS^®^ group the regular daily intake of 100 mg ASS^®^ was a necessary requirement. Underlying diseases requiring therapeutic or prophylactic anticoagulation were atrial fibrillation for all Marcumar^®^ treated patients (n = 20) and coronary heart disease (CHD; n = 27) or peripheral arterial disease (PAD; n = 3) for the ASS^®^ group. General exclusion criteria were defined as followed: attendance to other clinical trials, hereditary hemorrhagic diathesis, hereditary coagulopathy, pregnancy, HIV, hepatitis B or C infection, administration of anticoagulants of other specification or combined treatments of diverse oral anticoagulants, chemotherapy, cortisone administration, liver insufficiency, Quick value <15 %, Hb value <10 mg/dl or missing of informed consent. Age, body height (BH) and body weight (BW) were documented to calculate the body mass index (BMI) as well as the gender to choose matched volunteers. In addition, the blood parameters hemoglobin (Hb), platelet count, activated partial thromboplastin time (aPTT), INR and C-reactive protein (CRP) levels were determined. For analyzing the wound pads 20 ml whole blood samples were collected from each participant in S-Monovette^®^ 10 ml blood collection systems (Sarstedt AG & Co., Nümbrecht, Germany) containing 1 ml citrate solution (0.106 mol/l Trisodium citrate) and immediately processed as described below.

### Analysis of blood samples and specimen

Samples and specimen were prepared and analyzed according to a newly designed method which has been established, standardized and validated in terms of accuracy, precision, specificity, linearity and reproducibility using a standardized thrombin reference substance (Haemochrom Diagnostica, Essen, Germany), but not published by peer review to date. Briefly, the method for measuring blood coagulation is based on a chromogenic assay [23], at which the formation of thrombin, a key player within the coagulation cascade, causes the enzymatic cleavage of the chromogenic substrate S-2238 (CHROMOGENIX; Instrumentation Laboratory, Bedford, USA). The resulting colorimetric change corresponds with the clotting time which is further influenced by the use of different fleece wound pads. For this purpose, 20 ml of citrate buffered whole blood samples were centrifuged at 170×*g* for 15 min to obtain platelet-rich plasma (PRP). Sodium citrate of 2000 µl PRP was antagonized with 200 µl calcium chloride (2.27 mmol/l CaCl_2_; Carl Roth GmbH & Co. KG, Karlsruhe, Germany). Subsequently, 75 µl of antagonized PRP and 125 µl TRIS buffer were added to different specimen within a 48-well plate (Sarstedt AG & Co., Nümbrecht, Germany). A volume of 200 µl S-2238 working solution was added to each well and optical density (OD) measurements were commenced by spectrophotometry (EON™ Microplate Spectrophotometer; BioTek Instruments GmbH, Vermont, USA).

Spectrophotometry as part of the coagulation assay was performed in three phases: A main kinetics run measuring the extinction at 405 nm (reaction kinetics) and 750 nm (scattering correction) wavelength, as well as a pre- and a post-kinetics absorbance measurement at 900 nm (path length reference) and 977 nm (path length test) wavelength. One complete run covered triplicates of one patient. Pre- and post-kinetics measurements were conducted for later calculations of path length corrections which were necessary due to different fluid absorption capacities of the tested specimen, resulting in varying layer thickness of the examined blood samples. Graphs, test results and data reduction were calculated by the program Gen5™ 2.0 Data Analysis Software (BioTek Instruments GmbH, Vermont, USA) so that mean values and standard deviations (STD) were obtained.

### Statistical analysis

The study was initially designed to detect a difference of 0.9 STD in a two group comparison (α = 0.05, Power = 80 %; t test). This would require 20 subjects per group. Mean values (MV) and standard deviation (STD) for each parameter were calculated. Data were evaluated by one-way ANOVA using GraphPad Prism 6 software (GraphPad Prism, La Jolla, CA, USA). Multiple paired comparisons were corrected using the Tukey–Kramer method and differences considered to be statistically significant at *p* < 0.05.

## Results

### Physical features of the wound pads

**Fig. 2:**
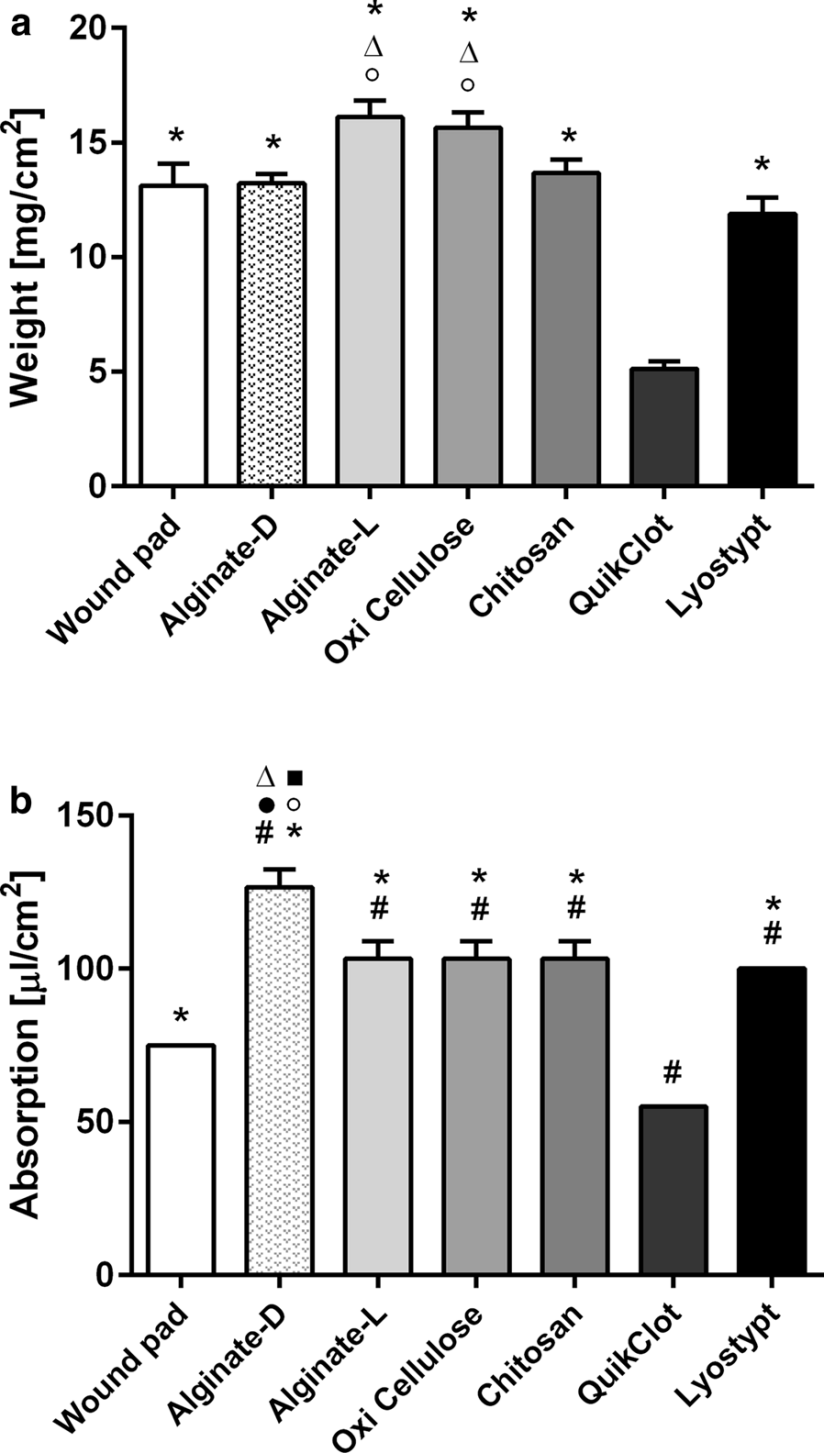
Dry weight (**a**; in mg/cm^2^) and absorption capacity (**b**; in µl/cm^2^) of the investigated specimens. Values are expressed as means ± STD. (^#^ *p* < 0.05 vs. standard wound pad; °*p* < 0.05 vs. alginate-l; ^●^ *p* < 0.05 vs. oxidized cellulose; ^ϫ^ *p* < 0.05 vs. Lyostypt^®^; *p < 0.05 vs. QuikClot^®^; ^■^ *p* < 0.05 vs. chitosan)

The weight of QuikClot^®^ (5.1 ± 0.33 mg/cm^2^) differed significantly from all other wound pads (Fig. 2a). Alginate-l showed the highest average weight (16.1 ± 0.73 mg/cm^2^) followed by oxidized cellulose (15.7 ± 0.68 mg/cm^2^). Alginate-l and oxidized cellulose had a significantly higher weight than alginate-d and Lyostypt^®^. For alginate-d and chitosan nearly the same weight was measured.

Regarding absorption capacity, QuikClot^®^ showed the lowest value with 55 µl/cm^2^ and hence differs significantly from all other analyzed pads including the standard wound pad (75 µl/cm^2^). Alginate-d on the contrary showed the highest absorption capacity (Fig. 2b), significantly higher than for all other tested dressings.

In terms of absorption speed, the standard wound pad showed the by far longest time needed (90 ± 11 s) to absorb 50 µl of whole blood. Chitosan and alginate-l needed 20 ± 3 and 16 ± 10 s respectively, which was significantly longer compared to other tested specimen. All other dressings were able to absorb 50 µl whole blood within 3 s.

### Adhesive strength, pH and temperature changes

**Fig. 3:**
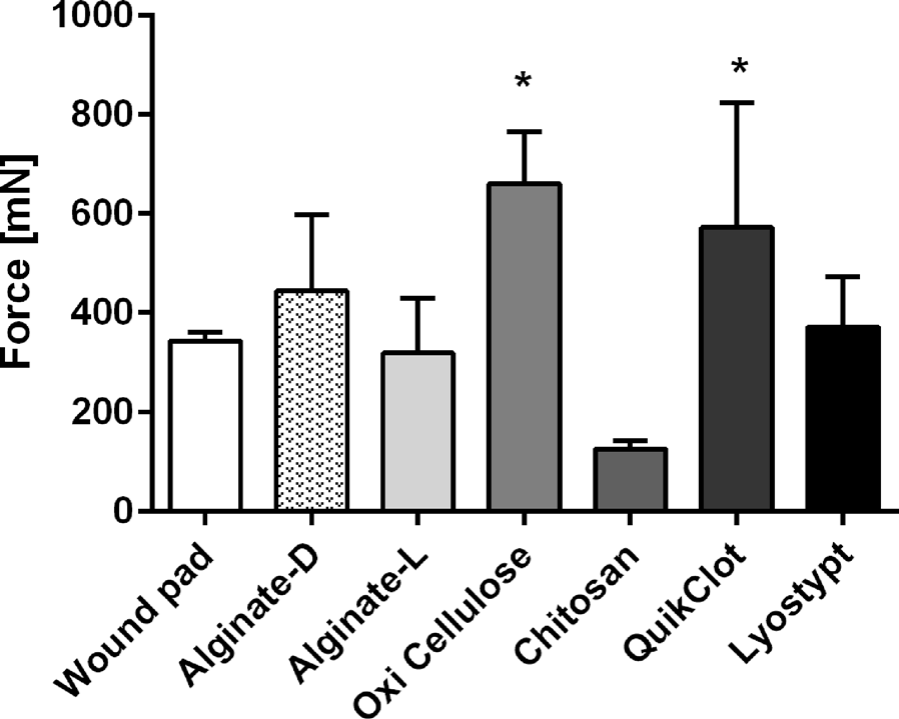
Force (in mN) needed to remove agglutinated specimens from porcine skin after 24 h. Values are expressed as means ± STD. (*p < 0.05 vs. chitosan)

Significantly less strength was necessary to detach chitosan (126.7 ± 16.1 mN) from porks’ skin compared to oxidized cellulose and QuikClot^®^ (660.0 ± 106.1 mN; 572.5 ± 251.0 mN) after 24 h (Fig. 3). Alginate-l (318.3 ± 154.9 mN) and the standard wound pad (342.5 ± 17.7 mN) also showed comparably little adhesion to the porcine skin. For Lyostypt^®^ and alginate-d a force of 371.7 ± 100 and 443.3 ± 154.9 mN was necessary for removal, respectively.

The pH of human FFP was significantly lowered by the alginate-d and alginate-l compared to the other pads at nearly all time points (Table 2). Within the first 30 min, oxidized cellulose induced a significantly higher drop in pH-value than QuikClot^®^ and chitosan. Lyostypt^®^ displayed an acidifying effect after 30 min (up to 90 min) with a significantly lower pH-value than chitosan and Quikclot^®^. The two latter dressings seemed to have no acidifying effect; their pH-values were in line with those of the control and the standard wound pad.

**Table 2:** pH value of different wound pads submerged in human fresh frozen plasma (FFP) of volunteers at baseline, after 15, 30, 60 and 90 min

	Control	Wound pad	Alginate-d	Alginate-l	Oxidized Cellulose	Chitosan	Quikclot^®^	Lyostypt^®^
Baseline	7.33 ± 0.03^b^	7.34 ± 0.03^b^	7.08 ± 0.04^a,c,d,e,f,g,h^	7.26 ± 0.08^e,f^	7.25 ± 0.04^e,f^	7.43 ± 0.04^b,c,d^	7.44 ± 0.06^b,c,d^	7.33 ± 0.05^b^
30 min	7.48 ± 0.02	7.47 ± 0.02^c^	7.35 ± 0.04^e,f^	7.29 ± 0.13^e,f,h^	7.4 ± 0.07^f^	7.53 ± 0.02^b,c,g^	7.57 ± 0.03^b,c,d,g^	7.36 ± 0.02^e,f^
60 min	7.57 ± 0.04^c^	7.56 ± 0.04^b,c^	7.2 ± 0.01^a,c,d,e,f,g,h^	7.38 ± 0.11^a,b,d,e,f,h^	7.53 ± 0.05^b,c^	7.61 ± 0.02^b,c,g^	7.65 ± 0.03^b,c,g^	7.4 ± 0.04^b,e,f^
90 min	7.64 ± 0.03^b,c,g^	7.63 ± 0.03^b,c,g^	7.23 ± 0.04^a,c,d,e,f,g,h^	7.47 ± 0.08^a,b,e,h^	7.55 ± 0.05^b,f^	7.66 ± 0.02^b,c,g^	7.7 ± 0.01^b,c,d,g^	7.5 ± 0.02^a,b,e,f,h^

Values are expressed as means ± STD

^a–h^Means differ significantly (*p* < 0.05, One-way ANOVA) to indicated superscripted dressings. ^a^ Wound pad, ^b^ alginate-d, ^c^ alginate-l, ^d^ oxidized cellulose, ^e^ chitosan, ^f^ Quickclot^®^, ^g^ Lyostypt^®^, ^h^ control

The temperature did not differ between the tested specimens and remained constant at 22.7 ± 0.05 °C in human FFP at all measured time points (data not shown). In addition, the pH-value of the untreated human FFP rose slightly during the 90 min measurement (7.33 ± 0.03–7.64 ± 0.02) while the temperature stayed steady.

### Patients and volunteers’ data

For all recruited patients blood test results of Hb, platelet count, aPTT, Quick value, INR and CRP were gathered (Table 3). Only the aPTT, INR and CRP values of the Marcumar^®^ group differed significantly from those of the untreated and ASS^®^ group. Regarding BW, BH and BMI no significant differences could be observed whithin the groups.

**Table 3:** Patients’ data and blood parameters

	Healthy volunteers	Marcumar^®^ patients	ASS^®^ patients
Platelet count (n/nl)	258.6 ± 77.87	239.3 ± 87.37	255.5 ± 11.4
INR	0.99 ± 0.15*	2.35 ± 0.67	0.98 ± 0.05*
aPTT (s)	30.14 ± 4.05*	45.9 ± 12.46	30.22 ± 6.24*
Hb (g/dl)	13.94 ± 1.18	12.94 ± 3.44	13.61 ± 1.26
CRP (mg/dl)	8.76 ± 7.52*	38.48 ± 12.87	5.97 ± 6.15*
Age (years)	62.4 ± 7.4	76.2 ± 9.1	68.3 ± 11.4
Body weight (kg)	82.4 ± 10.0	84.2 ± 19.9	86.8 ± 21.2
Body height (cm)	170 ± 1.0	171 ± 8.0	172 ± 9.0
BMI (kg/cm^2^)	28.08 ± 3.12	28.52 ± 4.91	29.93 ± 7.43
Gender (female/male)	10/10	13/7	16/14

Values are expressed as means ± STD

* p < 0.05 vs. Marcumar^®^

### Coagulation assay

**Fig. 4:**
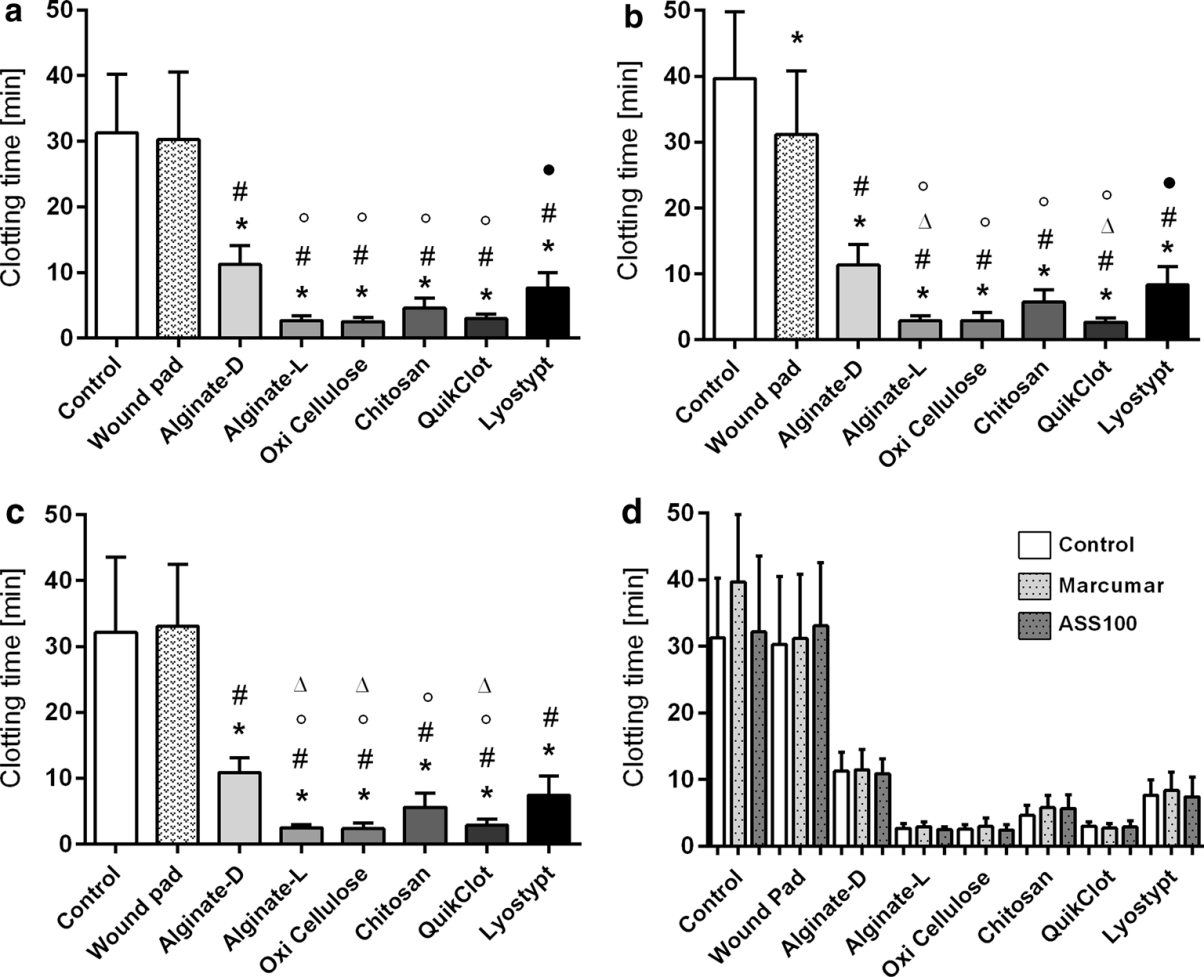
Clotting time (in minutes) induced by the different investigated wound pads and a control without specimen application. Results were assessed via spectrophotometric extinction measurement of thrombin activity. **a** Healthy volunteers, **b** Marcumar^®^ patients, **c** ASS^®^ patients, **d** summarized graphic depiction of all groups (statistics not reported). Values are expressed as means ± STD. (*p < 0.05 vs. control; ^#^ *p* < 0.05 vs. standard wound pad; °*p* < 0.05 vs. alginate-d; ^●^ *p* < 0.05 vs. oxidized cellulose; ^ϫ^p < 0.05 vs. Lyostypt^®^)

The spectrophotometric analysis of all tested wound pads showed a remarkable significant reduction in clotting time compared to the untreated positive control as well as to a standard wound pad (negative control) for all three groups (Fig. 4a–c). QuikClot^®^, oxidized cellulose and alginate-l demonstrated significantly faster clotting times than the materials chitosan or collagen (Lyostypt^®^). Of the latter, chitosan had the tendency to induce clotting faster than the established Lyostypt^®^. The coagulation onset of alginate-l is nearly the same (healthy: 2.46 ± 0.35 min; Marcumar^®^: 3.06 ± 0.45 min; ASS^®^: 2.40 ± 0.24 min) as that of the well known and already established QuikClot^®^ (healthy: 3.13 ± 0.39 min; Marcumar^®^: 2.51 ± 0.43 min; ASS^®^: 2.59 ± 0.51 min). In contrast, alginate-d manufactured by another company (Dr. Ausbüttel & Co., Witten, Germany) could not reach this level. However, its clotting induction time was still significantly shorter than that of the standard wound pad.

Comparison of the three groups revealed no significant difference in terms of clotting times of a specimen when exposed to Marcumar^®^, ASS^®^ or untreated blood samples. Results for clotting times are very equal for the individual pad, regardless of the studied group, while the control without specimen application showed a relevant difference (healthy: 30.41 ± 8.27 min; Marcumar^®^: 39.44 ± 9.51 min; ASS^®^: 32.56 ± 11.05 min; Fig. 4d).

## Discussion

With regard to demographic changes the percentage of patients with cardiovascular diseases such as essential hypertension, myocardial infarction, strokes, atrial fibrillation and heart valve replacements will rise in western countries [24]. Relating to this fact the number of patients in need of APT and ACT will increase rapidly [1]. Both therapies and their combinations reduce the likelihood of blood clot formation and thereby reduce the overall thromboembolic risk. However, this positive effect also faces the difficulty of impaired blood clotting which becomes dangerous in case of tissue injuries or surgical interventions.

While a decelerated coagulation has a lower impact in case of small and superficial wounds, it becomes an increasing risk after traumatic injuries. Still, minor injuries and the delayed coagulation is already disturbing for the individual, diminishes quality of life and might even become a burden. Furthermore, patients with cardiovascular diseases often show the same risk factors as stated for the development of chronic wounds like higher age, diabetes mellitus, arterial hypertension, obesity and tobacco use [25]. Such parallels might lead to the co-occurring of both medical conditions with the need for intermittent (surgical) local debridement in an anticoagulated patient. Hence, there is a need for wound dressings that promote clotting with all beneficial physical properties of a proper wound dressing and counter the ACT locally, without decreasing the overall systemic thromboembolic risk prophylaxis. In contrast to traumatic major bleedings, where some of the tested dressings are commonly used, the physical features of a plaster pad play an important role at superficial wounds: the pad has to be smooth, light, comfortable to wear, and especially non-adhesive. Complications from dressing changes such as reopening of the wound should not arise.

In order to compare the hemostatic potential of different specimen in vitro, a new method to measure coagulation induction by solid materials was developed. Tests such as chromogenic assays or platelet function (aggregometry) are well known and established to determine specific activities within the coagulation cascade [26, 27]. Due to poor standardization and different methods applied there is no established standardized in vitro method for measuring the hemostatic effect of materials such as fleece dressings or other wound pads. All of these procedures focus only on fluids with or without corpuscular fraction. The attempt to indirectly measure the hemostatic effect via thromboelastography (TEG) led to results which did not reflect the clinical results in terms of induction of hemostasis and clotting [28]. The newly developed coagulation assay applied in this study uses spectrophotometric determination of thrombin activity enabling a standardized, reproducible and unsusceptible in vitro method for the objective measurement of clotting time in solid materials and dressings. This facilitates the thorough in vitro evaluation of newly developed hemostatics prior to in vivo and clinical trials.

Several available topical agents such as fibrin sealants, silicates and others improve local hemostasis [29–31]. However, study designs were not comparable and most focused on major hemorrhage in surgical intervention, severe trauma or battlefield injuries. The prolonged bleeding of superficial everyday wounds are insufficiently analyzed [29, 32].

Wound pads with hemostatic features are well known from emergency and combat medicine with QuikClot^®^ Combat Gauze (Z-MEDICA, Wallingford, USA), Lyostypt^®^ (B. Braun Melsungen AG, Melsungen, Germany), Celox^®^ (MedTrade Products Ltd, Cheshire, UK), WoundStat^®^ (TraumaCure, Bethestta, MD, USA) and a dry fibrin sealant dressing (Ethicon^®^, Johnson and Johnson, Sommervilee, USA) as common representatives. According to the FDA classification (Food and Drug Administration, Dept. of Health and Human Services, USA) all pads, except the fibrin sealant, are class II products and therefore do not contain released substances.

QuikClot^®^ Combat gauze is composed of kaolin-impregnated rayon and polyester hemostatic dressing that advances coagulation by rapidly absorbing fluid. This results in an accumulation and concentration of cellular blood components and coagulation factors at the wound site. Although QuikClot^®^ demonstrated accelerated coagulation induction regardless of the applied ACT (Marcumar^®^, ASS^®^ or untreated), it showed poor results in terms of absorption capacities and was not able to absorb the necessary blood volume of acute bleeding superficial wounds. Due to the fact that QuikClot^®^ showed the second highest adhesive strength the risk of reopening of the wound upon removal is increased. Furthermore, the previously produced QuikClot^®^ based on zeolite derived from volcanic rock was replaced by the manufacturer in 2010 due to adverse effects, such as (micro-)embolism and tissue damage caused by an exothermic reaction [33, 34].

Oxidized cellulose, manufactured from wood pulp, consists of a polyanhydroglucuronic acid. Up to date, the exact mechanism of its hemostatic action is unknown. Presumably, clotting is supported by physical effects rather than interfering with coagulation cascade components [35]. It is mainly used in the surgical setting since it is absorbable and can therefore be left in place within body cavities. In this in vitro study oxidized cellulose had comparable hemostatic capabilities to QuikClot^®^ and was superior to Lyostypt^®^. Blood absorption capacity of oxidized cellulose was significantly better than for QuikClot^®^ indicating an advantage for the use as a superficial wound dressing. On the other hand its feature to dissolve as well as its strong adhesion might be disadvantageous for treating superficial wounds.

The hemostatic effect of chitosan which is a polysaccharide belonging to the group of biopolymers is not exactly known either. It has been discussed to induce vasoconstriction leading to a local accumulation of blood cells and clotting factors. Additionally, chitosan intensifies thrombocyte adhesion and aggregation at damaged tissue [36]. However, the efficacy of inducing and accelerating clotting is still being discussed, as several studies showed a significant reduction in bleeding and mortality [37–39] while others deny these effects [40, 41]. In terms of physical parameters, chitosan showed good results in absorption capacity and dry weight, but comparatively slow absorption speed. Regarding adhesion to porcine skin it demonstrated the lowest adhesion of all tested specimen. The clotting time was six-fold accelerated compared to alginate-d and even predominated the established collagen Lyostypt^®^. Thus, and due to good additional haptic abilities, chitosan might be suitable as a plaster wound pad, particularly for wounds with high exudation rates with the further option to control hemostasis.

Derived from bovine corium, microfibrillar collagen, such as Lyostypt^®^, induces hemostasis accompanied by low rates of tissue reaction and fast absorption capacities [42] and demonstrated hemostatic effects in animal studies [43, 44]. These effects are based on the promotion of platelet aggregation in which thrombocytes adhere to the collagen while undergoing a morphological change during the process of clotting. This results in an additional clot strengthening. Despite the coagulation promoting effect of Lyostypt^®^, nearly all tested specimen of the present study induced coagulation faster. Quikclot^®^ and alginate-l showed significantly faster clotting times for both groups with ACT. Even though good results of the adhesion and absorption tests indicate no concerns for the usage of Lyostypt^®^ as a plaster wound pad, its handling is difficult [43] and superior results reported in other studies [45, 46] could not be confirmed.

Alginate dressings are often used in the management of chronic wounds due to their bacteriostatic effect and high wound fluid absorption capability [47, 48] which is in line with the results of the present study. Due to limited information and evidence about the hemostatic potential of alginates, different kinds were tested in this study revealing variations in the range of ±15 % (alginate-d vs. alginate-l) in terms of absorption capacity. The known blood absorption capacities of alginates resulting in advanced coagulation [49] could be confirmed for alginate-l in the in vitro assay. The clotting time was equal to that of Quikclot^®^ or oxidized cellulose. Alginate-d however, showed comparatively poor results, but still significantly predominated the standard plaster wound pad. Furthermore, no disturbance of wound healing or other adverse effects have been reported for alginates [49]. The combination of all declared features of the alginate-l make it the first choice out of the investigated superficial wound dressings for the faster induction of coagulation combined with its favorable effects on promoting wound healing.

A very interesting result of this in vitro study is the fact that all analyzed superficial wound dressings reduced the clotting time to the same level regardless of the patients’ ACT (ASS^®^ or Marcumar^®^). In contrast, results from the controls of each group without any specimen contact showed distinct differences in clotting time indicating that the pads are capable of neglecting the anticoagulating effect of the investigated ACTs in vitro.

In terms of limitations to this study it needs to be emphasized, that the presented results represent a first in vitro exploration into the topic of hemostatic dressings for superficial wounds in anticoagulated patients. The results serve as a foundation for future in vitro and in vivo investigations to further support the reported findings in this study. Another limitation regards the tested ACTs in this study. Due to the predominant use of Marcumar^®^ and ASS^®^ in clinical practice in Germany these were chosen for preliminary examinations. In light of the present increase in the use of new oral anticoagulants (NOACs) such as direct factor Xa inhibitors (rivaroxaban) and P2Y12 antagonists (clopidogrel, ticagrelor) these medications still need to be evaluated in order to reach a more general conclusion on the treatment of superficial wounds of patients receiving ACT with hemostatic dressings. Such investigations, as well as first in vivo tests are currently in the process of planning. Regarding the study population the gender proportion in the Marcumar^®^ group was not balanced. Inclusion of patients treated solely with Marcumar^®^ proved to be difficult, since most patients received a double or triple ACT (additional application of antiplatelet drugs) which has been defined as an exclusion criterion. Also, analysis of patients blood parameters revealed significantly increased levels of aPTT and CRP in the Marcumar^®^ group. A possible explanation for elevated CRP levels could be preceding interventions, as no relevant underlying disease such as infection and no heparin treatment as explanation for elevated aPTT levels were reported in the investigated study groups. While aPTT level elevation under treatment with vitamin K antagonists have been reported previously [50], no causal correlation between Marcumar^®^ therapy and CRP elevation is known to date. Therefore, an influence of elevated CRP levels on the reported results in clotting times are not expected, but can not be entirely precluded either. Further research is needed to confirm that hemostatic dressings might provide an equal induction of clotting for the individual patient in superficial wounds, ruling out the disadvantage of different ACTs in local hemostasis.

## Conclusions

All analyzed superficial wound dressings reduced the clotting time to the same level regardless of the patients’ investigated ACT. Therefore, the hemostatic effect is based on physical (surface) and/or chemical (pH, temperature) interactions between the tested specimen and the patients blood, whereby the underlying mechanism of hemodilution (blocking platelet aggregation or interference with the coagulation cascade) seems secondary. With regard to the methods the spectrophotometry has the potential to be established as an objective method of choice for the investigation of clotting at or in solid materials.

In the light of the attained results the summarized evaluation of the dressings could be ranked as followed: Oxidized cellulose ≥ alginate-l > QuikClot^®^ > chitosan > Lyostypt^®^ > alginate-d > standard wound pad. Differences in prolonged bleeding times (Marcumar^®^ > ASS^®^ > untreated) could be equalized by applying a local hemostatic agent and therefore lower the risk of exaggerated bleeding in superficial wounds for each patient to the same level, as the demonstrated in vitro results suggest. Further in vivo investigations will be necessary to support these results. Alginate-L met the requirements of a superficial wound dressing best due to its fast coagulation induction combined with favorable physical properties, such as high absorption capacity and low tissue adhesion. Thus, it should be the first choice in the local treatment of “small” hemorrhage like bleeding skin wounds, in particular due to its additional positive effects in wound healing.
