# Erratum to: Comparison of hemostatic dressings for superficial wounds using a new spectrophotometric coagulation assay

**DOI:** 10.1186/s12967-016-0969-7

**Published:** 2016-07-25

**Authors:** Julian-Dario Rembe, Julia K. Böhm, Carolin Fromm-Dornieden, Nadine Schäfer, Marc Maegele, Matthias Fröhlich, Ewa K. Stuermer

**Affiliations:** 1 Institute for Research in Operative Medicine (IFOM), Witten/Herdecke University, Ostmerheimer Str. 200, 51109 Cologne, Germany; 2 Department of Traumatology, Orthopaedic Surgery and Sports Traumatology, Cologne-Merheim Medical Centre (CMMC), Witten/Herdecke University, Campus Cologne-Merheim, Ostmerheimer Str. 200, 51109 Cologne, Germany

## Erratum to: J Transl Med (2015) 13:375 DOI 10.1186/s12967-015-0740-5

After publication of our work [1] it was brought to our attention that the cited mechanism of action for the kaolin-impregnated gauze dressing QuikClot® needs to be extended. The statement regarding the products mechanism of action was based on the most recent publication regarding QuikClot® (Reference #20 in the article, Li et al. 2013), yet this article investigated an outdated formulation of the product based on zeolite instead of kaolin. The kaolin-impregnated gauze activates factor XII, initiating the intrinsic coagulation pathway [2]. Therefore, the following corrections are made:

1. In “Table 1 – Investigated dressings” the mechanism of action reading: “Unknown; presumably concentration of procoagulant factors due to water absorption and Ca^2+^ release [20]” for QuikClot® needs to be replaced with: “Activation of coagulation factor XII [20]”, whereby the reference Lamb et al. 2012 (here [2]) replaces #20 (Li et al. 2013) as reference for the mechanism of action.
2. In the discussion section page 7 of 10, line 15 the statement “… that advances coagulation by rapidly absorbing fluid.” needs to be extended by: “… and activates factor XII of the coagulation cascade.”

Corrections and extension of the above statements regarding the mechanism of action of QuikClot® does not alter the findings or conclusions of the original article.
